# Molecular composition of skeletal muscle in infants and adults: a comparative proteomic and transcriptomic study

**DOI:** 10.1038/s41598-024-74913-4

**Published:** 2024-10-03

**Authors:** Alexander Schaiter, Andreas Hentschel, Felix Kleefeld, Julia Schuld, Vincent Umathum, Tara Procida-Kowalski, Christopher Nelke, Angela Roth, Andreas Hahn, Heidrun H. Krämer, Tobias Ruck, Rita Horvath, Peter F. M. van der Ven, Marek Bartkuhn, Andreas Roos, Anne Schänzer

**Affiliations:** 1 Institute of Neuropathology, Justus-Liebig University, Giessen, Germany; 2 Leibnitz Institut für Analytische Wissenschaften-ISAS e.V., Dortmund, Germany; 3 Department of Clinical Neurosciences, School of Clinical Medicine, John van Geest Centre for Brain Repair, University of Cambridge, Cambridge, UK; 4 Department of Neurology, Charité - Universitätsmedizin Berlin, corporate member of Freie Universität Berlin and Humboldt-Universität zu Berlin, Berlin, Germany; 5 Department of Molecular Cell Biology, Institute for Cell Biology, University of Bonn, Bonn, Germany; 6 Institute of Pathology and Molecular Pathology, Bundeswehrkrankenhaus Ulm, Ulm, Germany; 7 Institute for Lung Health (ILH), Justus-Liebig University, Giessen, Germany; 8 Department of Neurology, Medical Faculty and University Hospital Düsseldorf, Heinrich-Heine-University Düsseldorf, Düsseldorf, Germany; 9 Department of Pediatric Neurology, Justus-Liebig University Giessen, Giessen, Germany; 10 Department of Neurology, Justus-Liebig University Giessen, Giessen, Germany; 11 Translational Neuroscience Network Giessen (TNNG), Justus Liebig University Giessen, Giessen, Germany; 12 Biomedical Informatics and Systems Medicine, Justus-Liebig University Giessen, Giessen, Germany; 13 Department of Pediatric Neurology, Centre for Neuromuscular Disorders, Centre for Translational Neuro‐ and Behavioral Sciences, University Duisburg‐Essen, Essen, Germany; 14 Children’s Hospital of Eastern Ontario Research Institute, Ottawa, ON Canada

**Keywords:** Muscle proteomics, Muscle transcriptomics, Mitochondria in aging, Bioinformatics in omics, Pathogenesis, Developmental biology, Paediatric research

## Abstract

To gain a deeper understanding of skeletal muscle function in younger age and aging in elderly, identification of molecular signatures regulating these functions under physiological conditions is needed. Although molecular studies of healthy muscle have been conducted on adults and older subjects, there is a lack of research on infant muscle in terms of combined morphological, transcriptomic and proteomic profiles. To address this gap of knowledge, we performed RNA sequencing (RNA-seq), tandem mass spectrometry (LC–MS/MS), morphometric analysis and assays for mitochondrial maintenance in skeletal muscle biopsies from both, infants aged 4–28 months and adults aged 19–65 years. We identified differently expressed genes (DEGs) and differentially expressed proteins (DEPs) in adults compared to infants. The down-regulated genes in adults were associated with functional terms primarily related to sarcomeres, cellular maintenance, and metabolic, immunological and developmental processes. Thus, our study indicates age-related differences in the molecular signatures and associated functions of healthy skeletal muscle. Moreover, the findings assert that processes previously associated solely with aging are indeed part of development and healthy aging. Hence, combined findings of this study also indicate that age-dependent controls are crucial in muscle disease studies, as otherwise the comparative results may not be reliable.

## Introduction

Skeletal muscles, being the largest tissue in the body, play a crucial role in physical activity and quality of life. Reduced muscle function is often linked to neuromuscular disorders, which can be caused by genetic or acquired factors in childhood or adulthood. It is worth noting that even healthy older individuals experience a decline in muscle function from the age of 50^1^. Sarcopenia is a muscle disease that can affect individuals of any age, not just older patients. It is identified by a decrease in skeletal muscle mass and strength, which leads to impaired muscle function^2^. However, it is not fully understood, whether the decline of muscle function in sarcopenia is primarily due to a pathological condition or to the advanced aging process ^1^.

Precise knowledge of the physiological functions of healthy muscles is crucial to comprehending changes in plasticity and adaptation to aging. Hypothesis-free comprehensive molecular studies provide an opportunity for a deeper understanding of mechanisms underlying skeletal muscle function and affected structures in health and disease^3^. However, the majority of studies describing molecular profiling of human skeletal muscle focus on adult subjects from 20 up to 80 years of age. Major molecular alterations in muscle aging start around the age of 60 years and include changes in energy metabolism, mitochondrial function, autophagy, immune cell regulation and contractile elements^4–10^ (Table 1). Healthy skeletal muscle biopsies derived from infants are rarely investigated and thus, regulatory mechanisms are poorly understood.

**Table 1:** Literature review on molecular studies of human skeletal muscle in aging.

	Number and age of probands (years)	Main findings: downregulation with age	Main findings: upregulation with age
Staunton et al., 2012	n = 8 47–62 76–82	• Glycolytic metabolism • Contractile elements • Fast fiber (type 2)	• Slow fiber (type 1) • Mitochondrial enzymes • Succinatdehydrogenase
Théron et al., 2014	n = 10 50–79 Females only	• Energy metabolism • Myofilament • Cytoskeleton	
Murgia et al., 2017	n = 8 22–27 65–75 Single fiber analysis	• Glycolytic enzymes in fast fibers (type 2) • Chaperones in fast fibers • Mitochondrial content • Proteasome activity • Actin and myosin	• Glycolytic enzymes in slow fibers (type 1) • Actin and myosin chaperones in slow fibers
Ubaida-Mohien et al., 2019	n = 58 20–87	• Mitochondrial proteins • Citric acid cycle • Glycolysis	• Proteins associated with immunity • Proteostasis and alternative splicing
Gelfi et al., 2006	n = 12 20–25 70–76	• Regulatory myosin light chains • Glycolytic capacity	• Myosin heavy chain isoforms 1 and 2A • Oxidative metabolism
Gueugneau et al., 2014	n = 24 48–61 76–82 Females only	• Glycolytic and mitochondrial energy metabolism	• Protection against mitochondrial oxidative stress • Heat-shock-proteins • Chaperones
Tumasian et al., 2021	n = 53 22–83	• Mitochondrial transcripts and proteins	• Transcripts and proteins associated with immunity

To address this gap of knowledge, and to improve the current understanding of age-dependent regulating mechanisms under physiological conditions, we aimed to compare skeletal muscle biopsies from young children and adults using a combination of molecular and morphological approaches.

## Materials & methods

### Subjects characteristics and description of biomaterial

We performed morphological characterization and comparative molecular profiling of skeletal muscle biopsies from pediatric and adult subjects that were recruited from a single center in Giessen (Germany). From all subjects, biopsies were collected for diagnostic purposes prior to inclusion in our study. Inclusion criteria comprised absence of neuromuscular disorders according to clinical examination, laboratory parameters, and electrophysiological analysis as well as absence of major pathological alterations in muscle biopsy samples. In infants, all biopsies were collected from the quadriceps femoris muscle (QFM). In adults the majority of muscle biopsies were also taken from the QFM (n = 6). Additional biopsies from deltoideus muscle (DM; n = 2), gastrocnemius (GM; n = 1) and intercostal muscle (IM; n = 1) were included.

### Sample processing

#### Morphological studies

Muscle biopsies were processed for routine diagnostics according to standard procedure on 6 µm frozen sections. All muscle biopsies were reevaluated including ultrastructural analysis by transmission electron microscopy (TEM) to exclude significant morphological alterations.

Immunohistochemistry was performed on 6 µm frozen sections using an automated BenchMark XT staining platform (Ventana, Heidelberg, Germany, ultraview universal DAB staining kit) with primary antibodies against MHCdevelopmental (Leica, NCL-MHCd), MHCneonatal (Leica, NCL-MHCn), MHCfast (Leica, NCL-MHCf) and MHCslow (Leica, NCL-MHCs). Fast- (type 2) and slow-twitch (type 1) muscle fibers were identified by staining for myosin heavy chain fast and slow isoforms (MHCfast and MHCslow). Morphometric analysis of fiber size diameter and the proportion of slow-twitch (type 1) and fast-twitch (type 2) muscle fibers was performed at all samples. MHCslow stained sections were digitalized using a Hamamatsu Nano Zoomer S360 (40 × objective lens). To measure fiber diameter, at least two representative regions of interest (ROI) of tangentially sectioned muscle fibers were selected using NDP.view2 software. The diameter of MHCslow positive (MHCslow +) and MHCslow negative (MHCslow-) muscle fibers within the ROI was measured. To determine the ratio of slow-twitch and fast-twitch muscle fibers, MHCslow + sections were analyzed using QuPath software 0.4.3^11^. At least 250 fibers were counted per sample. Statistical analysis and graphs were performed with GraphPad Prism software (v. 9.5).

#### Indirect immunostaining and microscopic analysis of skeletal muscle sections

Immunofluorescence staining was performed at muscle sections from an infant and an adult subject. After fixation with acetone (− 20 °C) for 10 min, 6 µm cryosections were air dried, rehydrated with phosphate-buffered saline (PBS) and incubated with blocking solution (10% normal goat serum (NGS) in PBS) for 45 min at 37 °C. Sections were incubated with primary antibodies (Filamin A/C, RR90 1:30; Xin A/B, XR1 1:50; XIRP2, 7700, 1:100)^12,13^ diluted in 1% bovine serum albumin (BSA) in PBS overnight at 4 °C. Sections were washed twice with PBST (0.5% Tween-20 in PBS) and once with PBS, and incubated with secondary antibodies (GAM IgA-Alexa 488 1:200. SBA 1040–31; GAM IgG1 Alexa 594 1:300, Jackson 115–585-205, GAR Alexa 647 1:500, Invitrogen A21245) diluted in 1% BSA in PBS for at least 2 h at 37 °C. Sections were washed three times in PBST, followed by a dip in double-distilled water, and mounted with Fluoromount-G (Invitrogen, Thermo Fisher Scientific, Dreieich, Germany). Microscopic analysis of immunostained sections was performed using an LSM710 or LSM 900 confocal laser scanning microscope (Carl Zeiss GmbH, Oberkochen, Germany).

### Transcriptomics

#### Extraction of total RNA from human skeletal muscle

From each skeletal muscle sample twenty 10 µm thick sections were provided for RNAseq analysis. Total RNA was isolated with the RNeasy® Mini Kit (Qiagen) following the manufacturer’s instructions including on-column DNA removal by DNase digestion using the RNase-free DNase set (Qiagen). Disruption and homogenization of muscle tissue slices were performed in 600 µl Buffer RLT containing DTT by passing the lysate through an RNase-free needle (20G) and syringe until a homogeneous suspension was obtained. Purified RNA was subjected to quality control by capillary electrophoresis (4200 TapeStation, Agilent).

#### Library preparation and RNA sequencing (RNA-Seq)

For genome-wide analysis of gene expression, RNA sequencing libraries from isolated mRNA were generated and sequenced by the Genomics and Bioinformatics platform of the Institute for Lung Health (ILH) at the Justus-Liebig-University (JLU) Giessen (Germany). A total amount of 25 ng of RNA per sample was used to enrich for polyadenylated mRNA followed by cDNA sequencing library preparation utilizing the Illumina®Stranded mRNA PrepKit (Illumina) according to the manufacturer’s instructions. After library quality control by capillary electrophoresis (4200 TapeStation, Agilent), cDNA libraries were sequenced on the Illumina NovaSeq 6000 platform generating 50 bp paired-end reads. Generally, we sequenced > 20 × 10^6^ reads per sample.

#### Proteomics

From each skeletal muscle sample twenty 10 µm thick sections were provided for mass spectrometry (MS) analysis. Muscle sample lysis involved the addition of 200 µl of lysis buffer containing 50 mM Tris–HCl (pH 7.8), 5% SDS, and cOmplete ULTRA protease inhibitor (Roche). The Bioruptor® (Diagenode) was employed for 10 min (30 s on, 30 s off, 10 cycles) at 4 °C to facilitate lysis. To ensure thorough lysis, an additional step involved sonication using an ultra-sonic probe (30 s, 1 s/1 s, amplitude 40%), followed by centrifugation at 4 °C and 20,000 g for 15 min. Protein concentration was determined using the BCA assay as per the manufacturer’s instructions. Disulfide bonds were reduced by adding 10 mM TCEP at 37 °C for 30 min, and free sulfhydryl bonds were alkylated with 15 mM IAA at room temperature in the dark for 30 min. For proteolysis, 100 µg protein from each sample underwent S-Trap protocol (Protifi) digestion with a trypsin to protein ratio of 1:20 for 2 h at 37 °C. Proteolysis was halted by acidifying the sample (pH < 3.0) with formic acid.

Complete digestion of all proteolytic samples was verified after desalting using monolithic column separation (PepSwift monolithic PS-DVB PL-CAP200-PM, Dionex) on an inert Ultimate 3000 HPLC (Dionex, Germering, Germany). This involved direct injection of 1 μg sample and a binary gradient (solvent A: 0.1% TFA, solvent B: 0.08% TFA, 84% ACN) ranging from 5 to 12% B in 5 min and then from 12 to 50% B in 15 min at a flow rate of 2.2 μL/min and at 60 °C. UV traces were acquired at 214 nm^14^.

#### DIA-LC–MS/MS analysis

All samples were analyzed using an UltiMate 3000 RSLC nano UHPLC coupled to a QExactive HF mass spectrometer, and the total amount of peptide applied was always 1 µg. Samples were first transferred to a 75 µm × 2 cm, 100 Å, C18 precolumn at a flow rate of 10 µl/min for 20 min. transferred, followed by separation on the 75 µm × 50 cm, 100 Å, C18 main column with a flow rate of 250 nl/min and a linear gradient consisting of solution A (99.9% water, 0.1% formic acid) and solution B (84% acetonitrile, 15.9% water, 0.1% formic acid), with a pure gradient length of 120 min (3–45% solution B). The gradient was applied as follows: 3% B for 20 min, 3–35% for 120 min, followed by 3 wash steps, each reaching 95% buffer B for 3 min. After the last wash step, the instrument was allowed to equilibrate for 20 min. MS data acquisition was performed in DIA (data independent acquisition) mode using an in-house generated spectral library. Each analyzed sample was mixed with an appropriate amount of iRT standard (Biognosys). Full MS scans were acquired from 300–1100 m/z at a resolution of 60,000 (Orbitrap) using the polysiloxane ion at 445.12002 m/z as the lock mass. The automatic gain control (AGC) was set to 3E6 and the maximum injection time was set to 20 ms. The full MS scans were followed by 23 DIA windows, each covering a range of 28 m/z with an overlap of 1 m/z starting at 400 m/z and acquired at a resolution of 30,000 (Orbitrap) with an AGC of 3E6 and an nCE of 27 (CID).

### Bioinformatic analysis

#### Data processing of transcriptomic data

For demultiplexing and the subsequent FASTQ file generation we used Illumina`s bcl2fastq (2.19.0.316). Primary processing of the sequencing reads, i.e. quality control, filtering, trimming, read alignment and generation of gene specific count tables was performed using the nf-core RNA-seq v3.7 bioinformatics pipeline (NEXTFLOW version 23.04.23^15^. The hg38 genome and gene annotation was used as downloaded from Illumina´s iGenome repository (https://support.illumina.com/sequencing/sequencing_software/igenome.html). As the corresponding annotations did not include genes encoded the mitochondrial genome, we performed an independent analysis using Ensembl annotation (release 111) for analysis of differential gene expression of mitochondrial genes. The pipeline run was performed with default parameters in docker mode. The resulting tables with raw read counts were imported into R where all down-stream processing was performed. In a separate analysis mitochondrial gene expression was aligned with the ENSEMBL gene annotation^16^.

The raw counts were processed using the DESeq2 package^17^ to normalize the data and to identify differentially expressed genes between adults and infants. To optimize noise and variability in the RNASeq data the adaptive shrinkage method “ashr” was used to estimate variance of gene expressions towards a common value to improve accuracy of downstream differential expression analysis. In order to visualize the consistency of the samples with regards to experimental variables the groups were analyzed using principal component analysis (PCA) and hierarchical clustering. Statistical significance was calculated via Wald test. Transcripts that showed a significant adjusted p-value (< 0.05) and a log2 fold change larger than 1 or less than -1 were interpreted as significantly differential expressed genes (DEGs).

#### Data processing of label-free proteomics

To identify significantly differential expressed proteins (DEPs), raw data acquired by nano-LC–MS/MS in DIA mode were imported into the Maxquant software (v2.0.3.0) and analyzed using a library-based search^18^. The standard significance levels FDR = 1% as well as the “matched between runs” option and label-free-quantification (LFQ) were utilized. The proteomic background is provided by the human proteome data from UniProt (https://www.uniprot.org/proteomes/UP000005640).

For further analysis complementary Perseus software (v1.6.15.0) was used^19^. Protein groups tagged with “only identified by site”, “Reverse” and “Potential contaminant” were filtered. Protein groups that were measured in at least 50% of biopsies in one group were further analyzed. Missing values were imputed from the normal distribution.

Statistical differences between the groups were analyzed using PCA and hierarchical clustering. Statistical significance was calculated via a two-sample t-test with an FDR < 0.05. Proteins that showed a significant adjusted p-value and a log2 fold change greater than 1 or less than -1 were interpreted as significant DEPs.

#### Enrichment analysis

Gene Ontology (GO) and Hallmark databases were used for annotation databases this analysis^20,21^. GO database provides a consistent vocabulary for annotating genes and gene products, focusing on their biological processes, molecular functions, and cellular components. The Hallmark Gene Set database categorizes sets of genes that define specific biological states or processes. While GO provides detailed annotations at various levels of biological function, Hallmark gene sets offer a more abstracted view, ideal for understanding broader biological themes and pathways. The Molecular Signatures Database (MSigDB) provided GO and HALLMARK pathway gene lists^21^. To interpret large lists of GO-Terms and to reduce their redundance we used the “rrvgo” R package^22^.

To identify regulated biological pathways between the two experimental groups, both overrepresentation analysis (ORA) and gene set enrichment analysis (GSEA) were utilized^23,24^. Overrepresentation enrichment analysis (ORA) was used to identify significant pathways, which are overrepresented among the DEPs and DEGs. GSEA allows to identify significantly enriched pathways based on pre-ranked gene lists. It enables to detect pathways based on all genes measured even if individual genes did not reach statistical significance. R package clusterProfiler was used for performing ORA and GSEA on the proteomic and transcriptomic datasets^23,24^.

### Graphs

The plots were visualized using the ggplot2^25^, Complex heatmaps^26^ and Enhanced Volcano R-libraries^26^.

### Mitochondrial assays

#### Quantification of mitochondrial DNA copy number (mtDNA CN)

The relative mtDNA copy number per cell was quantified by a multiplex Taqman qPCR assay (Bio-Rad 4,369,510), following a previously published protocol^27^. *B2M* and *MT-ND1* were used as nuclear and mitochondrial encoded reference genes, respectively. The primers used for the qPCR reaction were: B2M Fw-CACTGAAAAAGATGAGTATGCC, Rv-AACATTCCCTGACAATCCC; MT-ND1 Fw-ACGCCATAAAACTCTTCACCAAAG, Rv-GGGTTCATAGTAGAAGAGCGATGG. All samples were run in triplicates and replicates greater than 0.5 Ct difference were removed. The relative amount of mtDNA was calculated at the Ct value difference of *B2M* and *ND1*, where Delta *C*_t_ (ΔC_*t*_) equals the sample C_*t*_ of the mitochondrial gene (*MT-ND1*) subtracted from the sample C_*t*_ of the nuclear reference gene (*B2M*).

#### Long range PCR for mtDNA deletions

The presence of mtDNA deletions in the major arc (10 kb) of the mitochondrial genome was assessed by long-range PCR. Custom-made primers were used (Fw: 5’-CCCTCTCTCCTACTCCTG; Rev: 5’-CAGGTGGTCAAGTATTTATGG). PCR amplification was performed on a T100 Thermal Cycler (Bio-Rad) using 1 µl of DNA and PrimeSTAR GXL DNA Polymerase (Takara Bio Europe) according to the manufacturer’s recommendations. The PCR products were electrophoresed on a 0.7% agarose gel for 65 min. Statistical analysis and graphs were performed with GraphPad Prism software (v. 9.5.0).

## Results

### Subjects and skeletal muscle morphology

Eight young subjects aged 4–28 months (I 1–8) and ten adult subjects aged 19–65 years (A 1–10) were included in the study to decipher molecular differences underlying natural aging in skeletal muscle. All muscle biopsies were performed as a diagnostic tool for suspected neuromuscular or cardiac disease. At the histological and ultrastructural level, skeletal muscle biopsies did not demonstrate any pathological changes, and there was no evidence of myopathy in the course of examination. Clinical characteristics and morphological findings are summarized in Supplemental Table 1. Details of assignment to the analyzes performed are shown in Supplemental Table 2.

#### Immature muscle fibers are present only in infants

**Fig. 1:**
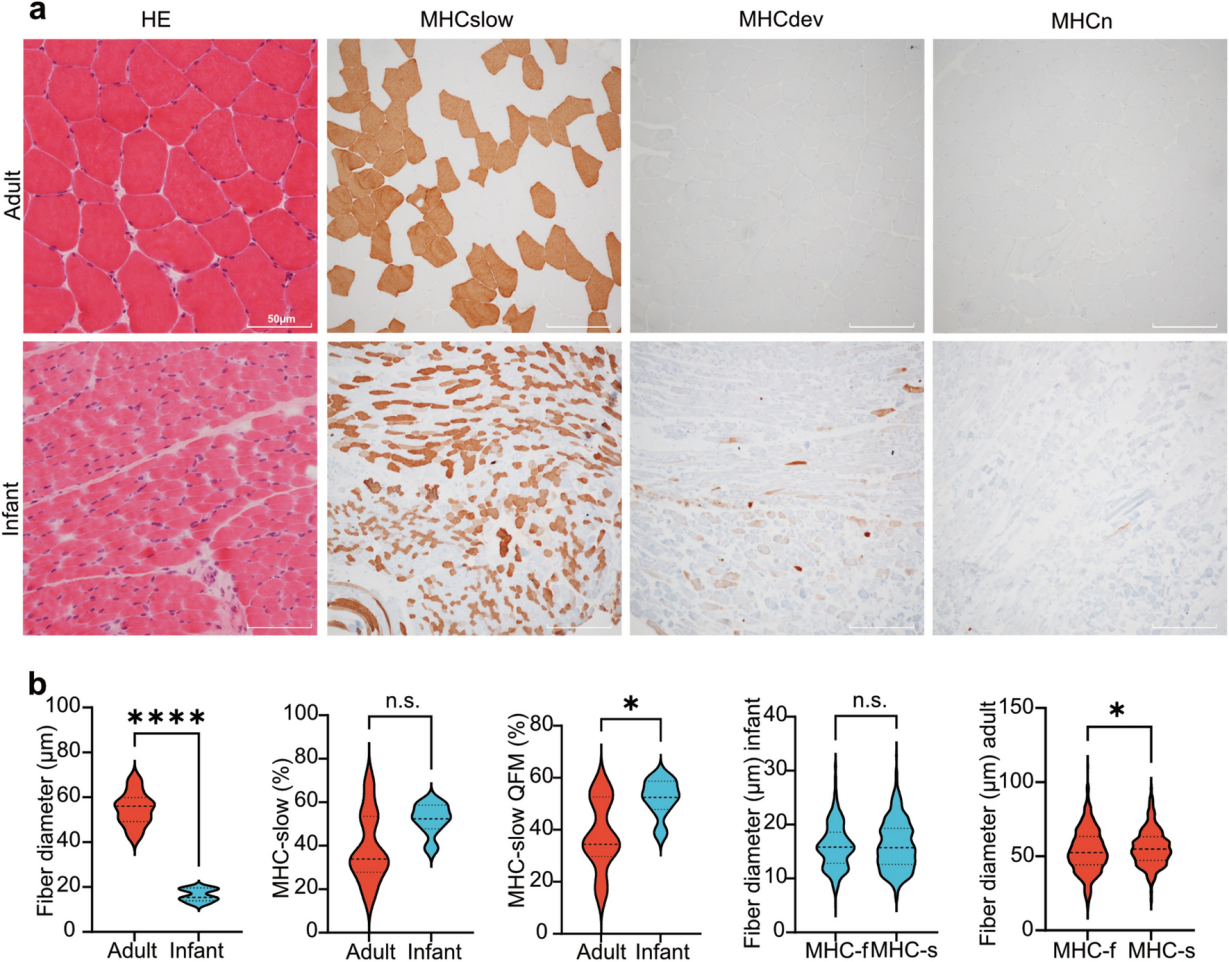
Morphometric analysis of muscle samples. (**a**) Representative histology of skeletal muscle biopsies from adult (A8, 40y) and infant (I2, 4mo) at HE stained cryosections showing larger muscle fiber diameter in adults. Immunohistochemistry staining against MHCslow detects type 1 fiber with predominance of type 1 fiber in adult and equal distribution in infant. Several MHCdev expressing and few scattered MHCn expressing fibers indicate presence of immature fibers only in the infant. (**b**) In adults, the mean diameter of muscle fibers is significantly larger compared to infants. Lower proportion of MHCslow stained fibers in adults compared to infants in all biopsies does not reach statistical significance. Analysis of quadriceps femoris samples (QFM) reveals a statistically significant higher number of slow fibers in infants. The mean diameter of slow and fast fibers do not differ in the infant group, whereas in adults slow fibers (type 1) are significantly larger compared to fast fiber. (*p < 0.05; **p < 0.01; **p < 0.001; ***p < 0.0001; n.s. = not significant). The violin plot reflect the median and interquartile range.

In younger subjects, the skeletal muscle fiber diameter increases with age until they reach the full size of 60 µm in adults^28^. Representative H&E stained of skeletal muscle biopsies from adult (A8, 40y) and infant (I2, 4mo) revealed smaller muscle fiber in infants compared to adults. Immunohistochemistry staining against MHCslow shows a predominance of type 1 fibers in specimen derived from adults and equal distribution of type1 and type 2 (MHCslow negative) in infants. Several MHCdev expressing and few scattered MHCn expressing fibers indicate immature fibers only in the muscle biopsies derived from infants (Fig. 1a). Quantification of myofiber diameter revealed significantly smaller fibers in the infant group (mean 16.36; range 12.5–19.6 µm) compared to adults (mean 54.81; range 42.9–67.6 µm; p < 0.0001) (Fig. 1b).

#### Age related distribution of slow (type 1) and fast (type 2) muscle fibers

In quadriceps muscle from pediatric subjects (aged 3-5y) and adults (aged 20–60 y), fast (type 2) muscle fibers are more frequent than slow (type 1) muscle fibers^29^. In adult subjects from 60 years of age on, type 1 fiber grouping appears, and range of diameter increases up to 12–55 µm^29,30^. In our study, analysis of fiber type distribution revealed a non-statistically significant (p = 0.064) higher number of slow fibers in infants of 52.23% (range 38.9–60.1%) compared to adults of 38.99% (range 24.9–68.3%). When only QFM samples were included, percentage of slow fibers was significantly higher in infants (52.3%; range 32.9–60.1%) compared to adults (37.9%; range 17.5–55.2%; p = 0.034). Analysis of the diameter of slow (16.21 ± 4.59 µm) and fast fibers (16.02 ± 4.19 µm) revealed no difference between both fiber types in the infant group (p = 0.56) across all samples. In contrast, in adults, slow fibers (55.86 ± 12.23 µm) were significantly larger compared to fast fibers (53.69 ± 14.55 µm; p = 0.018) (Fig. 1b).

### Transcriptomic analysis identifies distinct gene expression programs in young versus adult subjects

Transcriptomic studies in human skeletal muscle revealed alterations in expression of genes encoding for muscle maintenance proteins associated with aging^7^. Age-dependent up-regulation of transcripts referring to immune response pathways and down-regulation of mitochondrial metabolism have been described before in skeletal muscle from subjects aged 18 to 87 years^4,5,10,31^.

**Fig. 2:**
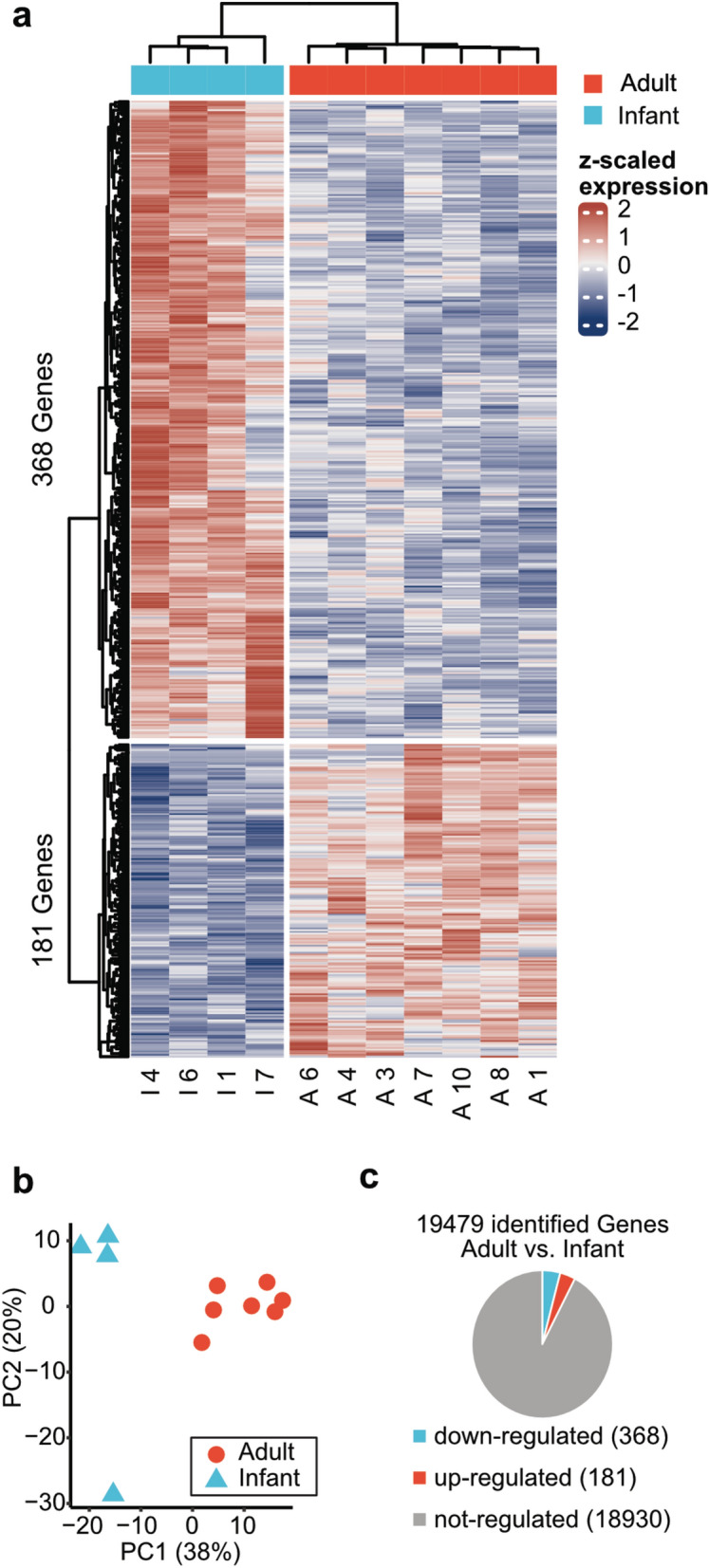
Transcriptomic analysis of skeletal muscle samples. (**a**) Hierarchical clustering shows systematic differences in gene expression patterns between infants and adults. Transcripts with adjusted p-value < 0.05 and a log2 fold change greater than 1 were plotted. (**b**) PCA of acquired data revealed systematic distinction between muscles samples derived from adults and infants. All 18,930 identified transcripts were used as a gene set in PCA analysis. (**c**) Pie chart with distribution of identified genes and DEGs in both groups.

In our study, RNA-seq was performed on muscle biopsies derived from 4 infants (mean age 15.9mo, range 7-28mo) and 7 adults (mean age 43.6y, range 30-56y). Hierarchical clustering and PCA of the transcriptomic data revealed two distinct groups (adult and infant) (Fig. 2a,b). Thus, our results indicate systematic differences in gene expression between infant and adult samples. RNA-seq analysis identified 19,479 expressed genes whereby the majority of the corresponding transcripts (n = 18,930) displayed no changes between both groups. 549 DEGs were identified with 368 down-regulated and 181 up-regulated transcripts in adults (adjusted p < 0.05 & log2-FC > 1) (Fig. 2c).

### Proteomic analysis reveals different protein abundances between adults and infants

**Fig. 3:**
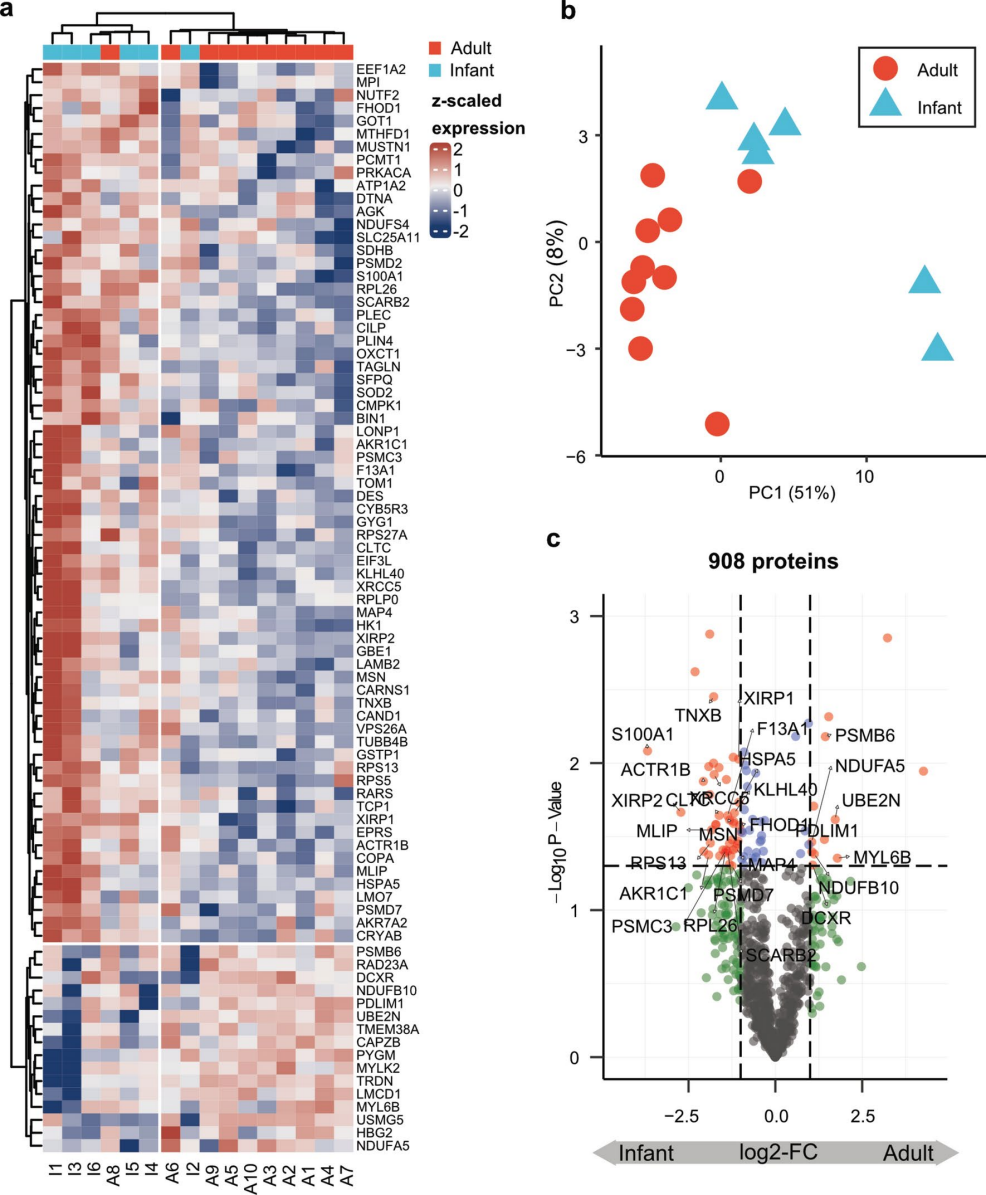
Proteomic analysis of skeletal muscle samples. (**a**) Hierarchical clustering of DEPs showing robust differences in protein expression patterns between infant and adult samples. (**b**) PCA of acquired data revealed systematic distinction between muscles samples derived from adults and infants. The 54 DEPs were used as a gene set for PCA analysis (**c**) Volcano plot of 908 identified proteins with 43 significantly down-regulated and 11 are up-regulated proteins in adult skeletal muscle. The x-axis displays the log2 fold change in expression and the y-axis the negative log10 of adjusted p-value. Proteins with p-value less than 0.05 and an absolute log2 fold change greater than 1 are colored red. Proteins that are further discussed in this paper are labelled with acronyms.

To unravel age-dependent changes in healthy skeletal muscle, unbiased proteomic profiling was performed on whole muscle protein extracts from 6 infants (mean age 16.17mo, range 4-28mo) and 10 adults (mean age 43.4y, range 19-65y). Proteomic findings were not normalized for fiber type ratio. In total, 2382 proteins were identified by MS, 908 of which were measured in at least 50% of the samples per group. The experimental groups were well-separated based on the hierarchical clustering analysis and PCA (Fig. 3a,b). Volcano plotting of 908 quantified proteins shows 43 significantly down-regulated and 11 are up-regulated proteins in adult skeletal muscle (Fig. 3c; Supplemental Table 3).

Analyzes of a possible effect of muscle biopsy localization did not reveal significant differences between the groups, as samples did not group according to the specific origin of the respective muscle biopsy specimen (Supplemental Fig. 1a,b).

#### Intersection of transcriptomic and proteomic data

Only 3 significant intersecting regulated genes were detected in both transcriptomic (DEGs) and proteomic (DEPs) datasets. A simulation approach was used to estimate the probability of observing an overlap of three or more significant genes between RNA-seq and mass spectrometry (MS) datasets by random chance resulting in an empirical p-value of 0.17. A low coverage of transcriptomic and proteomic data has been described in other studies before^32^. This might be related to regulative processes at the level of mRNA translation or a low correlation between the half-lives of proteins and mRNAs^33^ (Supplemental Fig. 2).

### Pathway analysis of transcriptomic and proteomic data

To elucidate the functional impact of differentially abundant transcripts and proteins on biochemical processes, ORA and GSEA were performed using the GO and Hallmark databases. Transcriptomic and proteomic data were processed separately. ORA provides insights into the functions of highly differentially expressed gene products, whereas GSEA encompasses trends across all measured transcripts and proteins. Analyzes yielding numerous regulated GO terms were clustered using the REVIGO TreeMap to enhance the readability of the results and are summarized in the next paragraphs^22^.

#### Structural and developmental proteins

**Fig. 4:**
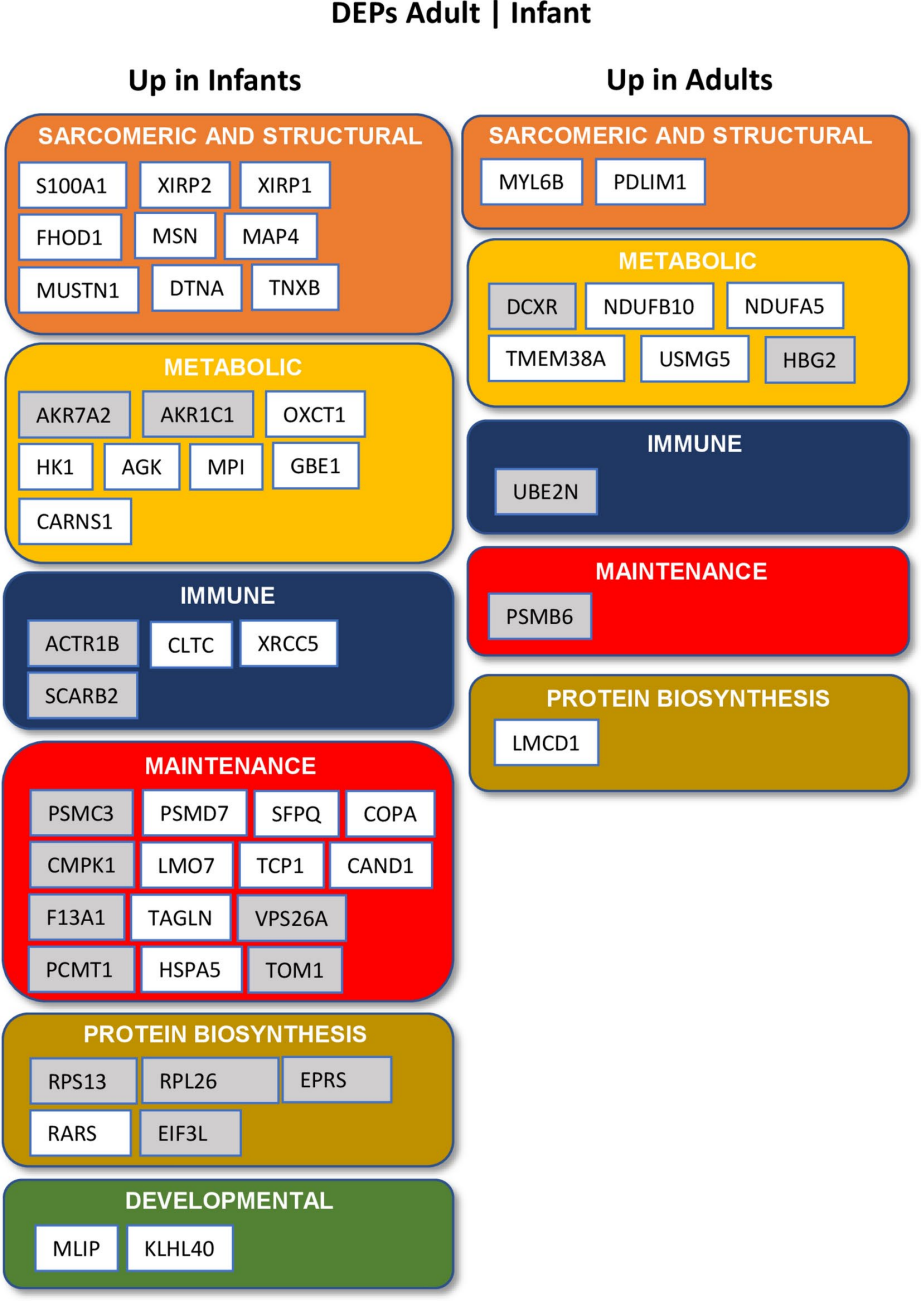
Overview of DEPs in adults compared to infants associated to biological functions. Grey boxes indicate that the protein has previously not been reported to be associated with skeletal muscle tissue (unknown muscle function).

Sarcomeric and cytoskeletal proteins present the major proportion in the skeletal muscle proteome^28,34^. In our study, transcriptomic analyzes showed down-regulation of GO cellular component terms “actin cytoskeleton” and “extracellular matrix organization” in adults (Supplemental Fig. 3). In older subjects, downregulation of myofilament and cytoskeleton-associated proteins account for approximately 20–30% of DEPs^6,10^. These data are in line with our observation that approximately 21% of down-regulated DEPs in adults attributed to myofibrils and other structural proteins (Fig. 4, Supplemental Table 3).

**Fig. 5:**
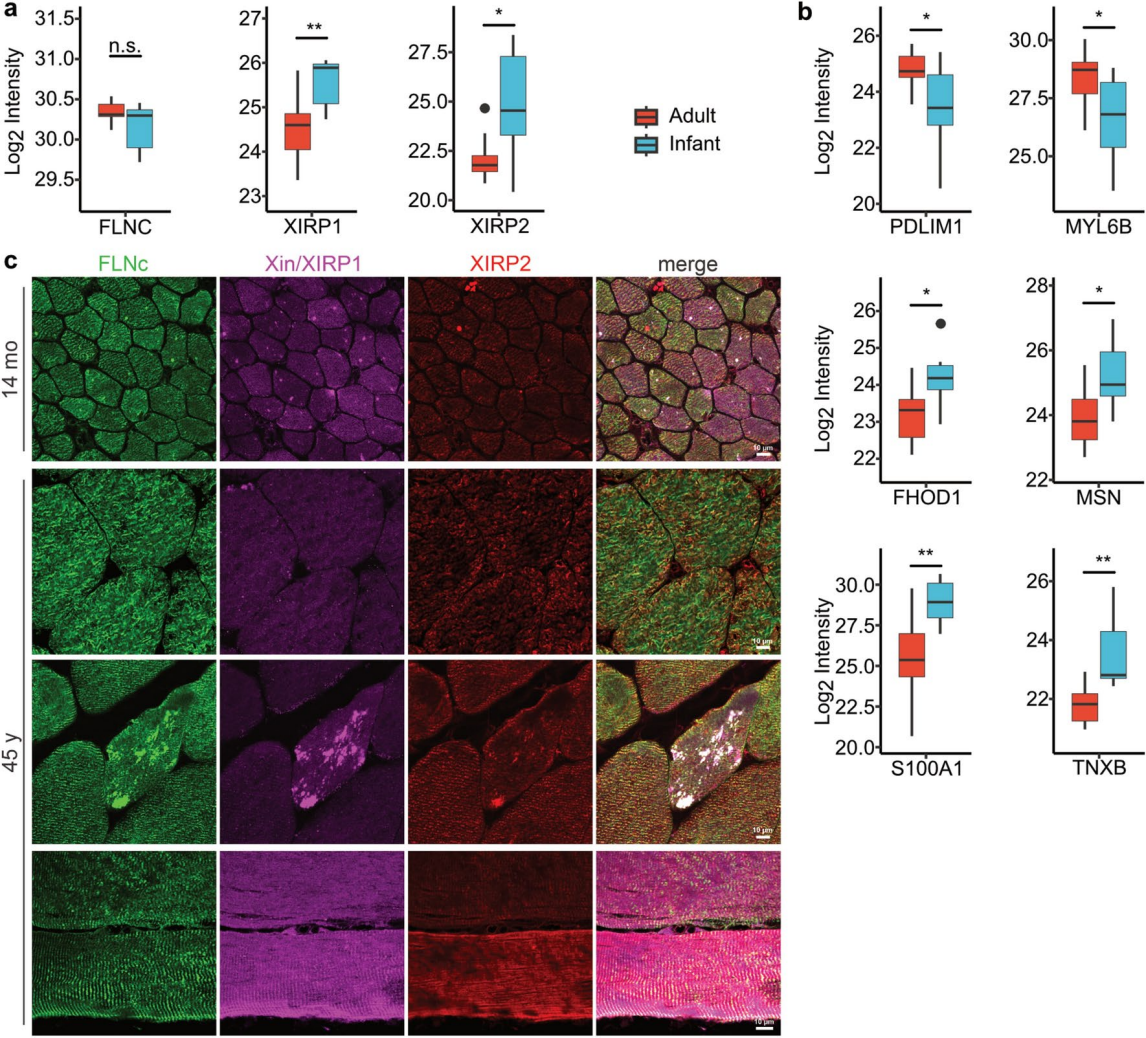
Structural and developmental proteins (**a**, **b**) DEPs associated with skeletal muscle structure and development. The boxes show the median and interquartile range. Outliers are indicated by black dots within the plot. Significant DEPs were defined with an adjusted p-value < 0.05 and a log2 fold change > 1. (**c**) Immunolocalization studies on cryosections of skeletal muscle biopsies from a 14 mo and a 45 y old individual with antibodies directed against filamin C (FLNc), Xin/XIRP1 and XIRP2. Some microlesion were detected by filamin C and Xin/Xirp2 in the adult sample. The different expression levels for Xin (Xirp1) and Xirp2 observed in mass spectrometry could not be validated by immunofluorescence assays, probably because of low their expression levels.

On proteomic level, the sarcomeric protein FLNC was not differentially regulated, whereas XIRP1 and XIRP2, which is mainly found in type 2 fibers, were down-regulated in muscle biopsies from adult individuals compared to such derived from infants (Fig. 5a)^35^_._ PDLIM1 and MYL6B, proteins mainly expressed in type 1 fibers were up-regulated in our adult samples^35^. Moreover, associated with the GO term “molecular function—actin binding”, FHOD1 and MSN were down-regulated in adults. Moreover, S100 calcium-binding protein A1 (S100 A1) and the extracellular matrix (ECM) protein tenascin-X (TNXB) were significantly down-regulated in our adult muscle samples (Fig. 5b). This is in line with the observation that TNXB and S100A1 are highly expressed in skeletal muscle during development^36,37^.

Immunofluorescence studies on representative sections from an infant and an adult subject showed strong expression of filamin C in the muscle fibers of both individuals. Filamin C is mainly localized in Z-discs that are clearly visible upon staining of longitudinal sections of the adult patient, but also in the somewhat oblique sections of the infant. Although our proteomics data showed increased expression of both Xin/XIRP1 and XIRP2 in young muscle fibers, which is in line with a function in muscle development, our immunostaining did not detect a clearly different staining intensity. However, some microlesion were detected by filamin C and Xin/Xirp2 in the adult sample that appear also in healthy skeletal muscle fibers upon muscle activity^12^ (Fig. 5c).

Beside other developmental processes the WNT signaling pathway was down-regulated in skeletal muscle of adults (Supplemental Fig. 3). Disruption of the WNT signaling pathway can lead to severe developmental disorders and impaired muscle homeostasis^38^. DEPs associated with developmental processes include MLIP and KLHL40, both are down-regulated in adults (Fig. 4, Supplemental Table 3). MLIP is required for myoblast differentiation into myotubes possibly acting as a transcriptional regulator of the myogenic program^39^. Diseases associated with MLIP include myopathies with myalgia and rhabdomyolysis and elevated baseline serum creatine kinase^40^. KLHL40 protein belongs to the superfamily of Kelch-repeat-containing proteins, striated-muscle-specific proteins involved in muscle development and function. Mutations in KLHL40 are associated with nemaline myopathy^41^.

#### Metabolic processes

**Fig. 6:**
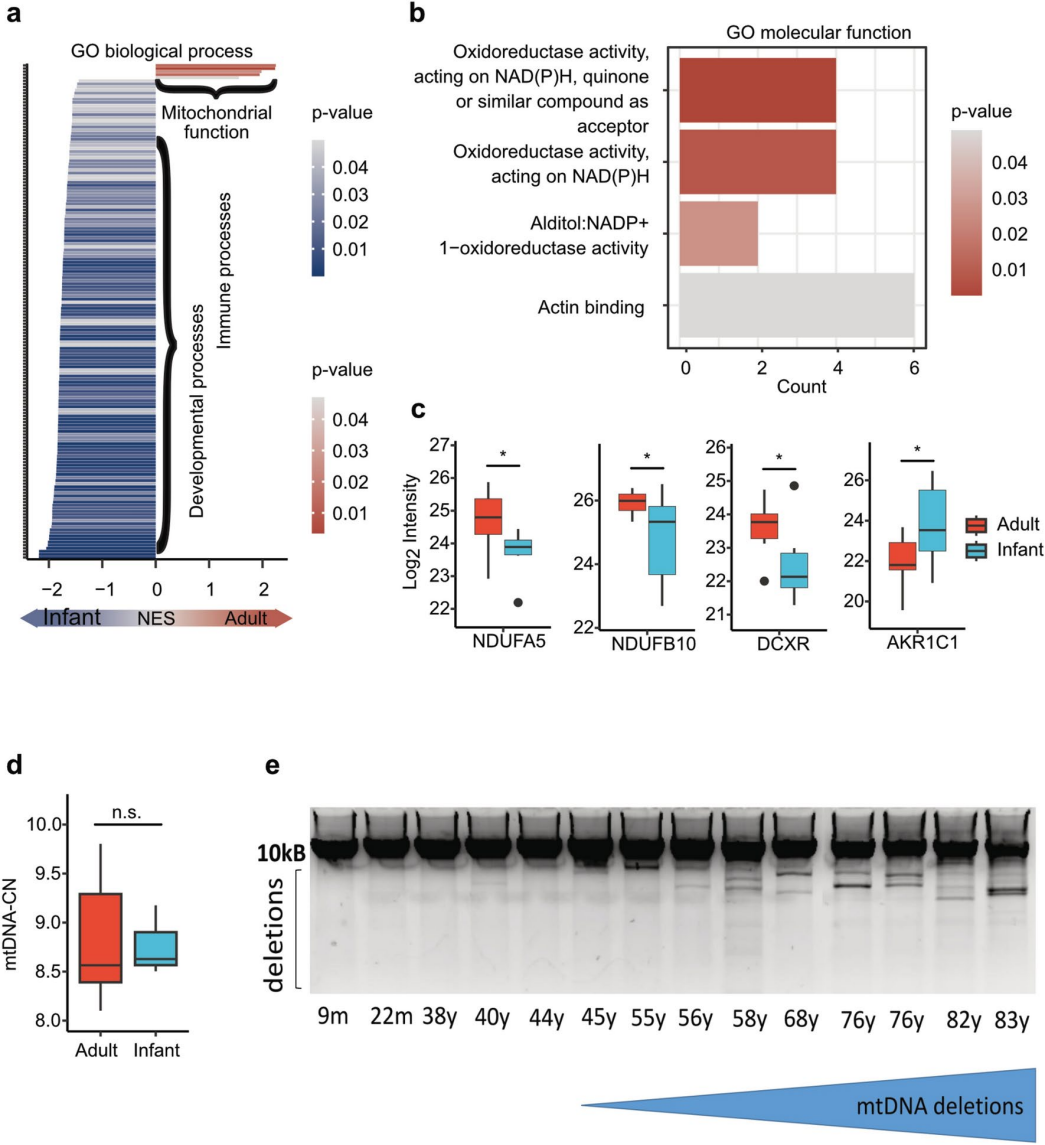
Metabolic and mitochondrial function. (**a**) With GO category “biological process” 214 deregulated terms were identified at transcriptomic analysis. Mitochondrial function was up-regulated in adults. Terms describing immune processes and developmental processes were up-regulated in infants. (**b**) With GO category “molecular function” four deregulated terms concerning NADP + dependent metabolism and actin binding were identified between both groups by proteomic analysis. (**c**) DEPs associated with metabolic and mitochondrial function. The boxes show the median and interquartile range. Outliers are indicated by black dots within the plot. Significant DEPs were defined with an adjusted p-value < 0.05 and a log2 fold change > 1. (**d**) Analysis of mitochondrial DNA copy number (mtDNA-CN) showed no significant difference between adult to infant samples (p > 0.05). (**e**) Detection of mtDNA-deletions by long range PCR showed alterations in individuals aged older than 55 years.

Skeletal muscle aging is associated with down-regulation of proteins involved in energy metabolism including mitochondrial respiration^4,5,9,10^. In transcriptomic analysis using GO category “biological processes” 214 terms were identified (Fig. 6a). Seven out of eight up-regulated terms in adults concern “mitochondrial gene expression” or “cellular respiration”. This is also highlighted in a REVIGO Treemap representation indicating only these two categories as upregulated (Supplemental Fig. 3). Separate analysis of mitochondrial gene expression using ENSEMBL annotation showed that only MT-ND6, encoding NADH dehydrogenase subunit 6, was slightly up-regulated in adults (Supplemental Fig. 5). In our proteomic dataset, terms from the GO category “molecular function”, especially those related to NAD(P)H + oxidation and reduction, were differentially regulated between adults and infants (Fig. 6b). DEPs associated with metabolic processes account for approximately 26% of all DEPs. In particular DEPs associated with these processes NDUFA5, NDUFB10 and DCXR, were up-regulated and AKR1C1 was down-regulated in adults (Fig. 6c, Fig. 4, Supplemental Table 3).

#### Age depended increase of mtDNA deletions from the age of 55 years

Several studies have investigated age-dependent changes of mtDNA copy number (mtDNA-CN). Whereas mtDNA-CN is decreased in blood samples from subjects aged 50 -70 years, data from mtDNA-CN of skeletal muscle show either no age related reduction, or increased levels in aged subjects^42^. So far, younger subjects have not been included in these studies. In our study, we analyzed mtDNA-CN from 3 infants (age 4-28 m, mean = 13 m) and 7 adults (age 30-56y, mean = 47.85y). Comparing the infants with adults, no significant difference of mtDNA-CN was detected in skeletal muscle samples (p = 0.85) (Fig. 6d). However, additional long-range PCR studies accompanied by the inclusion of muscle samples from 4 older subjects aged 76-83y unveiled an age depended increase of mtDNA deletions from the age of 55y on (Fig. 6e, supplemental Fig. 4).

#### Immune system

**Fig. 7:**
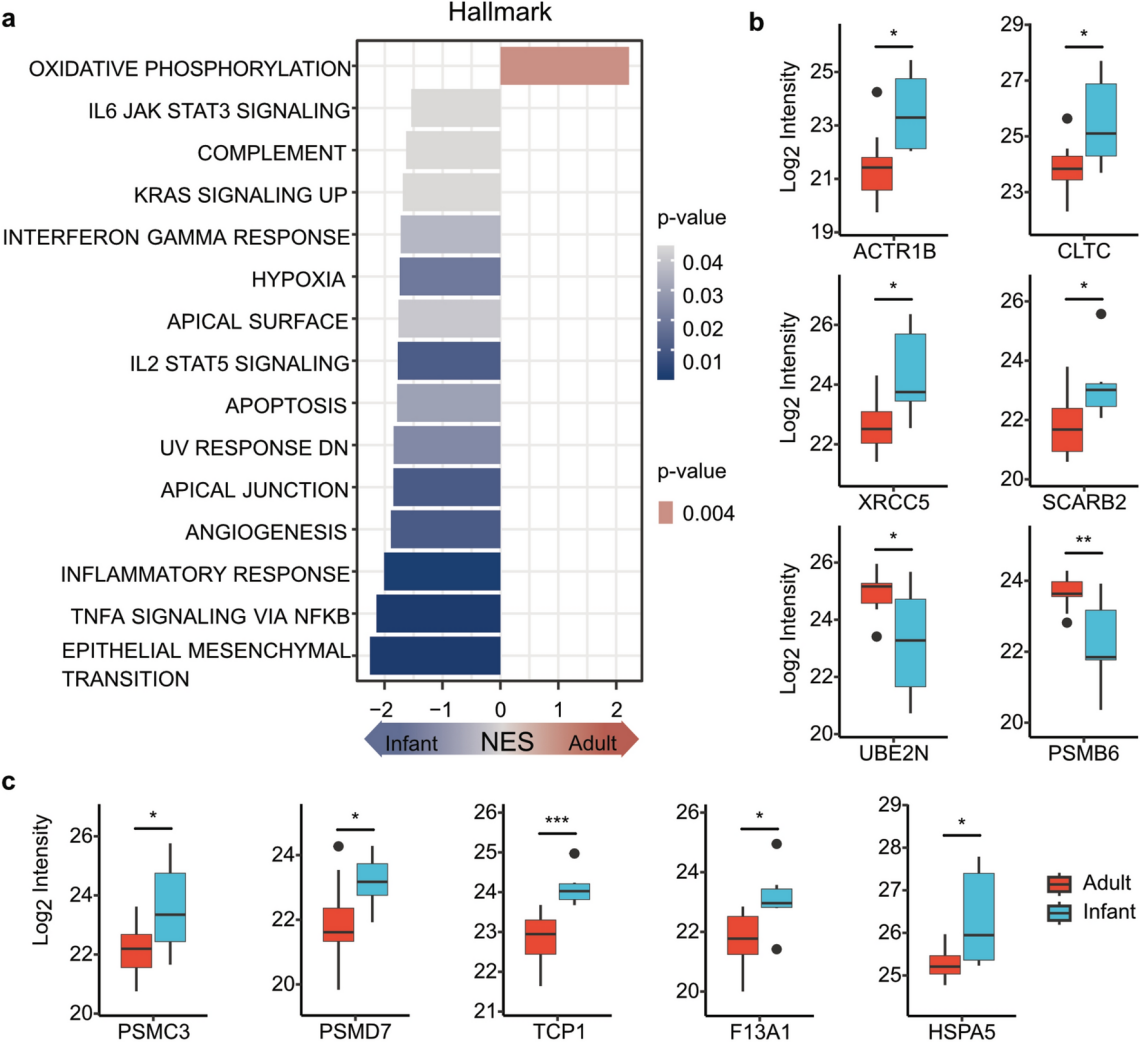
Immunologic and developmental function. (**a**) Transcriptomic analysis reveals 15 deregulated hallmark gene sets between infant and adult skeletal muscle. Only oxidative phosphorylation is up-regulated in adults. Immune response and development gene sets are up-regulated in infants. (**b**, **c**) DEPs associated with skeletal muscle maintenance and immune system. The boxes show the median and interquartile range. Outliers are indicated by black dots within the plot. Significant DEPs were defined with an adjusted p-value < 0.05 and a log2 fold change > 1.

Skeletal muscle aging is associated with up-regulation of proteins associated with immunity^4^. Our transcriptomic analysis revealed 15 deregulated hallmark gene sets with down-regulation of immune response and development and up-regulation of oxidative phosphorylation in adults (Fig. 7a). In our dataset, DEPs associated with immune processes account for approximately 11% of all DEPs. Proteins associated with immune processes ACTR1B, CLTC, XRCC5 and SCARB2 were down-regulated in adults, whereas UBE2N was up-regulated in adults (Fig. 7b, Fig. 4, Supplemental Table 3).

#### Muscle maintenance and cell homeostasis

Protein processing and clearance as well as cell hemostasis are altered in older age^10,35^. In our study, DEPs associated with muscle maintenance processes were mainly down-regulated in adults and account for approximately 26% of all DEPs. In particular proteasomal proteins PSMC3 and PSMD7, chaperone proteins associated with cell homeostasis TCP1 and F13A1 and heat shock protein HSPA5 were downregulated in adults. PSMB6 was upregulated in adults (Fig. 4, Fig. 7c, Supplemental Table 3). In contrast, transcriptomic analysis did not detect any pathways associated with protein processing and clearance processes and cell hemostasis.

#### Protein biosynthesis

Age-related loss of muscle strength is likely due to impaired muscle protein synthesis with ribosome biogenesis playing a major role in controlling muscle mass^43^. In line with these findings, our study showed down-regulation of ribosomal proteins (RPS13 and RPL26) in adults (Fig. 4, Supplemental Table 3). In contrast, transcriptomic analysis showed up-regulation of cytosolic small ribosomal subunits (Supplemental Fig. 3).

**Fig. 8:**
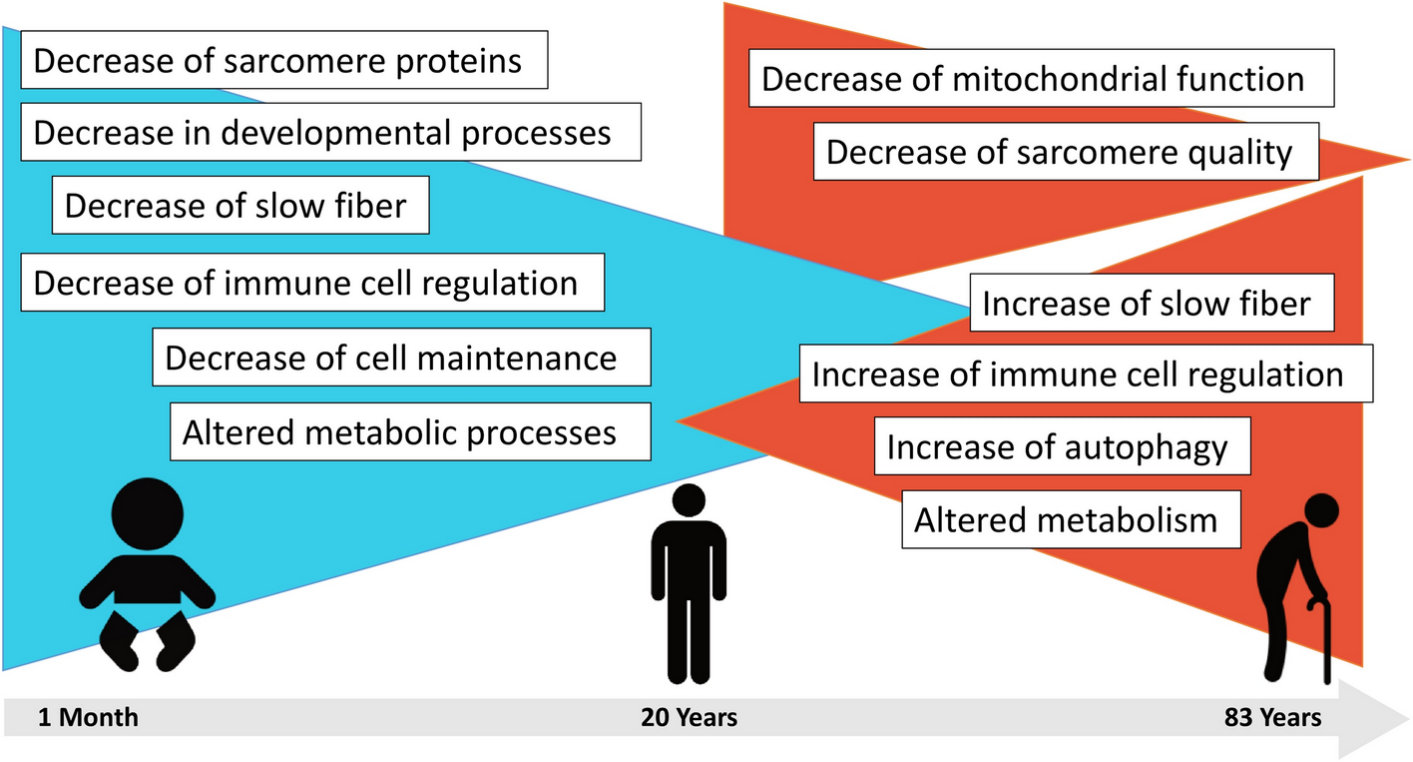
Summary of transcriptomic and proteomic findings in infants and adults in our study in comparison to those published in adult aging (Staunton et al. 2012, Théron et al. 2014; Murgia et al. 2017; Ubaida-Mohien et al. 2019; Gelfi et al. 2006; Gueugneau et al. 2014; Tumasain et al. 2021).

The summary of the major findings of our study, in comparison to previously published data concerning adult aging, is presented in Fig. 8.

## Discussion

The skeletal muscle fiber has a high capacity of plasticity and adaption to inner and outer stimuli and influences^44^. Skeletal muscle represents a major proportion of the total tissue mass of the human body, and impairment of muscle function in older age or disease leads to a reduced mobility and quality of life. To better understand and differentiate aging in healthy muscle, complex molecular studies are mandatory. Thus far, molecular analyzes of muscle throughout human life and during aging have been performed only on skeletal muscle samples from adult subjects aged 20 years and older, while younger subjects have not been included^4,6–10,32,35^. Therefore, our comparison represents an extended analytical approach and may shed light on the molecular processes underlying physiological changes in life and upon natural aging of skeletal muscle in humans.

To systematically address this lack of knowledge, we here present—to the best of our awareness for the first time—results of (combined) transcriptomic and proteomic analyzes along with a morphological characterization and molecular genetic studies of mtDNA, performed on infant muscle biopsies compared to adult biopsies.

Unbiased transcriptomic and proteomic profiling hold the potential to monitor thousands of transcripts and proteins in single experiments. Thus, we applied these technologies to obtain comprehensive insights into the molecular processes underlying natural aging of human skeletal muscle on the transcription and protein level. Hereby, data intersection enables to draw conclusions whether changes in protein levels are based on regulation of gene transcription or rather related to altered protein activity and/or stability.

In both, transcriptomic and proteomic analyzes, hierarchical clustering and PCA of obtained data indicated systematic differences between muscle samples derived from adults and infants. These findings indeed suggest the need for a deeper analysis of age-dependent molecular processes contributing to muscle function.

Skeletal muscle consists of two main muscle fiber types (slow type 1 and fast type 2), which have defined tasks and unique proteomic compositions. Postnatal growth and adaptation of skeletal muscle is modulated by muscle activity and complex interactions between chromatin and the transcription machinery. These processes largely regulate the remodeling of pre-existing fibers^45^. The distribution and size of muscle fibers are dependent on age with a type 2 predominance in children older than three years of age and adults. Our study revealed a differential distribution in infants and young children (4-28 months) with equal distribution of type 1 and type 2 fibers whereas in adults, predominance of fast type 2 fibers could be confirmed. The majority of studies analyzing muscle fiber transformation were performed in animal models. In human, muscle activity mainly effects muscle fiber transformation from slow type 2A to type 2B fibers in adults, which does not explain the different fiber type distribution in infants and adults^46^. However, altered innervation during development may play an important role in the determination muscle fiber type composition^47^. In infants, diameter of fast and slow fibers were similar, while in adults, type 2 fibers were significantly smaller compared to type 1 fibers. These findings are in line with the observation that size and volume of type 2 fibers gradually decrease with advanced age^48^. Interestingly, endurance exercise in the elderly is associated with muscle fiber adaption resulting in a transition from type 2 to type 1 fibers^49^.

In agreement with other studies, our proteomic profiling revealed down-regulation of XIRP1 and XIRP2 in adults, which is mainly found in type 2 fibers^35^. However, we could not confirm these mass spectrometric findings by immunfluorescence studies: XIRP1 and XIRP2 staining intensities were not clearly different between young and adult fibers. This might be caused by the rather low expression level of the protein. Dependent on the fiber type, XIRP2 is, for example, expressed at levels between 0.05 and 1.5% of the level of filamin C and in other cases proteomic analysis did not even detect any XIRP2 at all in the sarcoplasm of normal muscle fibers^50^, however this might be caused by different analytical approaches (data-dependent (DDA) versus data-independent (DIA) acquisition modes) applied in this study. Hence, this immunostaining finding also indicates that mass spectrometry-based protein quantification as a sophisticated analytical DIA-based approach is required to detect these subtle proteinogenic changes. PDLIM1 and MYL6B, mainly expressed in type 1 fibers, were up-regulated in adults^35,51^. However, data on fiber type dependent expression of sarcomeric proteins are until now only provided for muscle samples derived from adult individuals. In this context it is worth noting that proteins predominantly expressed in one fiber type may have differential functions such as development and regeneration (XIRP2), cytoskeleton organization (PDLIM1)^51^, and muscle growth and maintenance (MYLB6)^52^. These data are consistent with findings obtained by immunohistochemical approaches which revealed that infant skeletal muscle expresses neonatal and developmental myosin, and that fetal myosin is down-regulated from age of 4–6 month on^53^. Additionally, skeletal muscle development markers S100 and MAP4 are down-regulated in adults, highlighting stronger developmental capacity in infant muscle^54^. Furthermore, the ECM protein tenascin-X, which is highly expressed during skeletal muscle development^37^, was down-regulated in our adult samples. Moreover, our microscopic and proteomic findings are in part supported by our transcriptomic results that unraveled the down-regulation of transcripts encoding for proteins belonging to pathways associated with muscle cell development in adults.

In our study, proteins associated with sarcomere function and other structural proteins mostly showed reduced expression in adults compared to infants. This finding is in line with phenotypes observed during aging, which is associated with loss of muscle mass. The overlap to similar molecular processes taking place in sarcopenia underlines the challenge to molecularly dissect natural muscle aging and sarcopenia^4,6,10,48^. Consequently, our combined data suggest that processes being associated with sarcopenia may also determine healthy aging.

Differences of protein abundances in adult aging is strongest between young adults (20-34y) and old adults (> 80y) with decrease of proteins implicated in muscle contraction, muscle architecture and mitochondrial function in the elderly^4^. In older subjects, energy metabolism and mitochondrial proteins are known to be downregulated and to contribute to sarcopenia^4,10,35^. Our study indicated that genes and proteins associated with mitochondrial processes were upregulated in adults compared to infants although mitochondrial DNA levels showed no difference of mtDNA-copy number between adults and infants. Interestingly, our studies focusing on deletion events of mitochondrial DNA that included additional muscle samples from four older subjects up to 83 years, revealed an age-dependent increase of mtDNA-deletions from the age of 55 years on. These data highlight that alteration of mitochondrial DNA, and thus function might contribute to skeletal muscle aging in subjects from 55 years and older^35,55^. However, the finding of increased abundance of mitochondrial metabolism relevant proteins such as NDUFA5 and NDUFB10 (encoded by mtDNA) may accord with the identified subsequent changes of mtDNA stability most likely reflecting a cellular attempt of mitochondrial maintenance.

Enhancement of low-grade inflammation and deregulation of immunological processes contribute to loss of muscle mass and function in older age^4,56^. Additionally, skeletal muscle can serve as an endocrine organ, secreting myokines including interleukins (IL) such as IL6, IL7 and IL15, which is disturbed when muscle mass is reduced^57^. Loss of muscle mass and function is associated with a reduced physical activity, which in turn leads to an altered immune response. Our omics analyzes revealed that pathways associated with immune processes are indeed downregulated in adults. Interestingly, these pathways included IL6, IL2 and TNFα signaling mediated processes and interferon (IF) γ response. Hence, these findings indicate that a lower developmental capacity of myofibers might be associated with reduced immune processes.

It is known that in older age (> 70 years), heat shock protein and chaperone expression decreases, which hints towards disturbed capability of cellular protein maintenance and homeostasis^10,35^. Of note, our study confirmed a downregulation of chaperones and heat shock proteins in adults compared to infants. Additionally, differential regulation of proteasome associated proteins such as PSMC3 and PSMD7 highlight the role of age dependent mechanisms in protein clearance and are consistent with the observation that proteolytic capacity of the cellular proteasome declines with aging^36,58^.

Moesin (MSN) belongs to the ezrin-radixin-moesin (ERM) family of proteins that play structural and regulatory roles in the assembly and stabilization of plasma membrane interactions through their ability to interact with transmembrane proteins and the cytoskeleton^59^. In skin, MSN expression decreases with increasing age^60^, while in skeletal muscles MSN is expressed by myofibroblasts and might contribute to the fibrotic reaction in muscular dystrophies^61^. In our samples, MSN is downregulated in adults, rather indicating a loss of capacity for structural homeostasis in skeletal muscle fibers in older age than an antagonization of fibrotic remodeling based on our microscopic findings which did not reveal profound fibrotic remodeling.

Approximately 30% of the proteins with differential expression in infant and adult skeletal muscle have not yet been linked with a specific function in skeletal muscle. The functions described so far are based on other tissues, which might differ from their functions in skeletal muscle. Our findings not only indicate a possible role of these “unknown” proteins in age related processes in skeletal muscle but may also have implications for understanding the underlying mechanisms for muscle development and related disorders. Since we have not localized these proteins in muscle sections, we cannot assign whether these proteins are expressed in muscle fibers, or in other cells of the muscle tissue.

In the last years, the number of studies in muscular disorders including comparative proteomics to identify underlying mechanisms of muscle pathology has increased considerably. This development is most likely based on the enormous power of proteomic profiling in unveiling molecular mechanisms in an unbiased manner especially in combination with other omics techniques^62^. However, due to its limited availability, in particular studies on skeletal muscle tissue from infant patients are rare. Our study emphasizes, that age-matched control samples are absolutely necessary, especially when analyzing very young probands. Therefore, our data set will be useful for further studies of molecular profiling in pediatric muscular disorders. Hereditary myopathies often present a highly variable phenotype in early and late onset, even when associated with similar genetic background. Our findings in infant skeletal muscle samples highlight a specific molecular profile and morphology which may contribute to the muscular and clinical phenotype of muscular disease.

Our combined proteomic and transcriptomic data indicate a dysregulation of molecular determinants modulating natural aging of skeletal muscle including the altered abundance of structural proteins including sarcomeric proteins, altered abundance of proteins involved in the regulation of immune cells, changes in expression of proteins crucial for protein processing and clearance as well as changes in abundances of proteins playing different roles in metabolic activities of muscle cells (Fig. 8). In the latter context, it is important to note that although no significant changes in mtDNA copy number were identified, skeletal muscle from individuals aged 50 years and onwards presents with an increase of deletions within the mtDNA, a molecular finding, which most likely impacts on mitochondrial activity and maintenance and thus on overall metabolic processes in aged muscle cells. Overall, our combined omics findings are in line with results of previous studies on older individuals (listed in Table 1) and support the concept that changes in muscle cell metabolism and the contractile apparatus are key drivers of muscle aging.

### Limitations

A limitation of our study is the small number of muscle samples included and lack of muscle biopsies from older children from 3 years of age on. In addition, the findings in adult subjects could not be calculated in further age subgroups due to limited sample size. Muscle biopsies were taken for diagnostic reasons and even if we ruled out pathological effects on skeletal muscle by laboratory, electrophysiological and morphological analysis, individual skeletal muscle alterations cannot be excluded with absolute certainty. Although some of the infants showed neurological symptoms such as hypotonia and developmental delay, our molecular analyzes showed clear differences between molecular signature in the skeletal muscle in children and adults, underlying age dependent molecular regulation. We have analyzed the samples retrospectively. Unfortunately additional phenotypical data as body mass index or physical activity were not available. Due to the limited samples size of muscle tissue obtained from infants, additional quantitative analysis of proteins (e.g. western blotting) or RNA (e.g. qRT-PCR) could not be performed toward validation of our omics findings.

## Conclusions

In summary, our findings present a molecular analysis of skeletal muscle from young compared to adult probands. We identified genes and proteins that are differentially regulated depending on age, which highlights key molecular mechanisms for increasing our knowledge and understanding of the mechanisms underlying development and aging of human skeletal muscle. Our findings underline that age-dependent processes in older subjects already can be determined in younger subjects and suggests that processes underlying in sarcopenia may be advanced processes of healthy aging. The functional characterization of unknown muscle proteins may be helpful in further studies of skeletal muscle plasticity and muscle diseases at different ages.

## Supplementary Information

Supplementary Information.

## Data Availability

The mass spectrometry proteomics data have been deposited to the ProteomeXchange Consortium via the PRIDE partner repository with the dataset identifier PXD049424 ^63^. Raw and processed RNA-seq data in mESCs and MuSCs are available in the NCBI Gene Expression Omnibus (GEO), under accession number GSE 257,558.

## Abbreviations

DEP: Differential expressed protein
DEG: Differential expressed gene
DM: Deltoideus muscle
GM: Gastrocnemius muscle
GO: Gene ontology
GSEA: Gene set enrichment analysis
IM: Intercostal muscle
LFQ: Label-free-quantification
mo: Month
mtDNA-CN: Mitochondrial DNA copy number
MS: Tandem mass spectrometry
ORA: Overrepresentation analysis
PCA: Principal component analysis
QFM: Quadriceps femoris muscle
