# Supplementary material for: Molecular composition of skeletal muscle in infants and adults: a comparative proteomic and transcriptomic study
Source: Sci Rep. 2024 Oct 3;14:22965. doi: 10.1038/s41598-024-74913-4 (PMC11450201; doi:10.1038/s41598-024-74913-4)
Supplement: Supplementary file 1 — Supplementary Information. [file 41598_2024_74913_MOESM1_ESM.pdf]

Supplemental data

Molecular composition of skeletal muscle in infants and adults: a comparative proteomic and transcriptomic study

Alexander Schaiter[1], Andreas Hentschel[2], Felix Kleefeld[3,4], Julia Schuld[5], Vincent Umathum[1,6], Tara Procida-Kowalski[7], Christopher Nelke[8], Angela Roth[1], Andreas Hahn[9], Heidrun H. Krämer[10,11], Tobias Ruck[8], Rita Horvath[3], Peter F.M. van der Ven[5], Marek Bartkuhn[7,12]#, Andreas Roos[13,14]#, Anne Schänzer[1,11]#*

Supplemental Figure 1

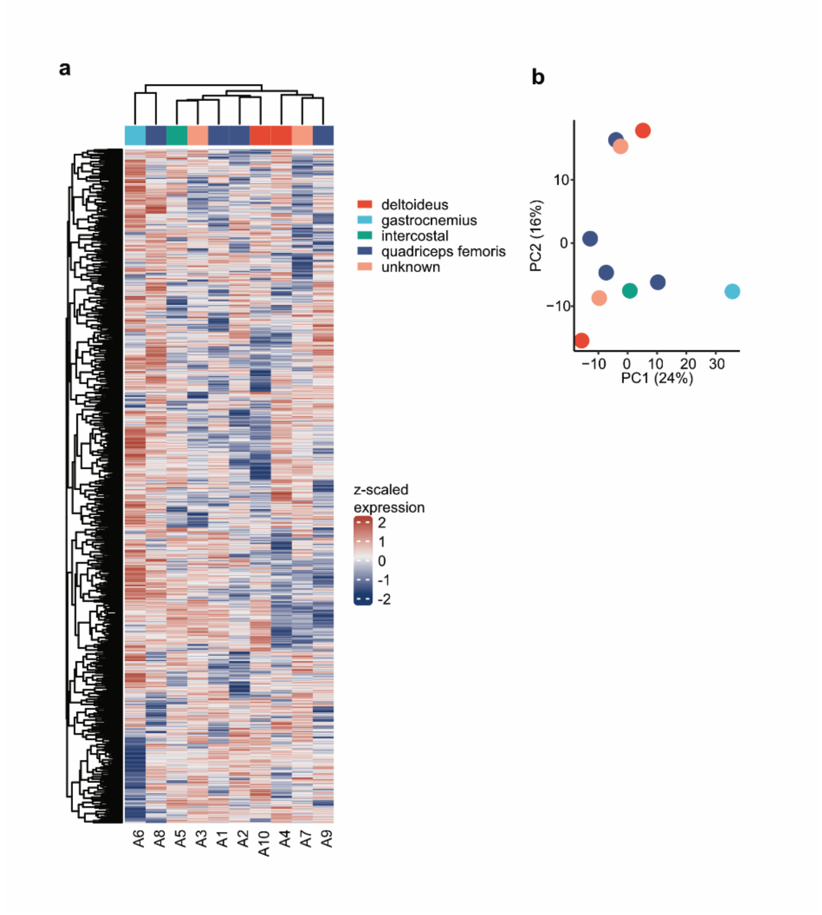

Supplemental Figure 1. Proteomic analysis of adult skeletal muscle samples according to site of biopsy. (a) Heatmap shows no apparent patterns of protein expression between samples in relation to site of biopsy. (b) PCA indicates no systematic differences of distinct groups of different sites of biopsy.

Supplemental Figure 2

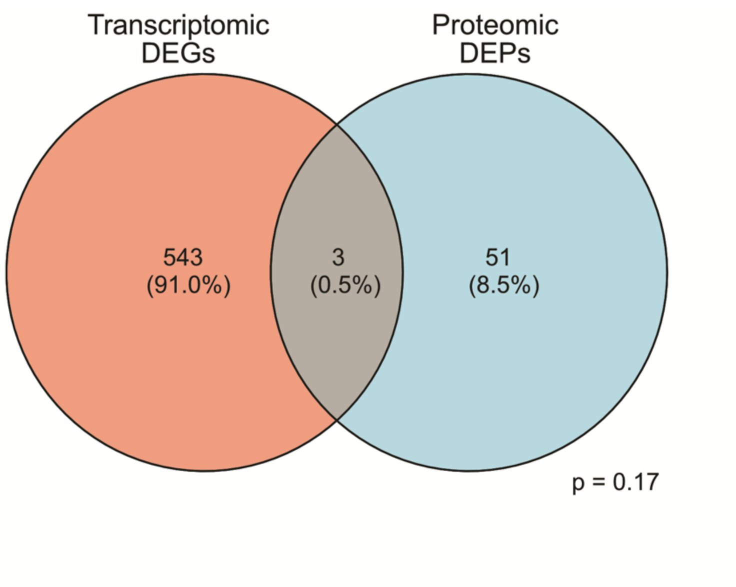

Supplemental Figure 2 Intersection of DEGs and DEPs depicted as a Euler-diagram. A simulation approach was used to estimate the probability of observing an overlap of three or more significant genes between RNA-seq

Supplemental Figure 3

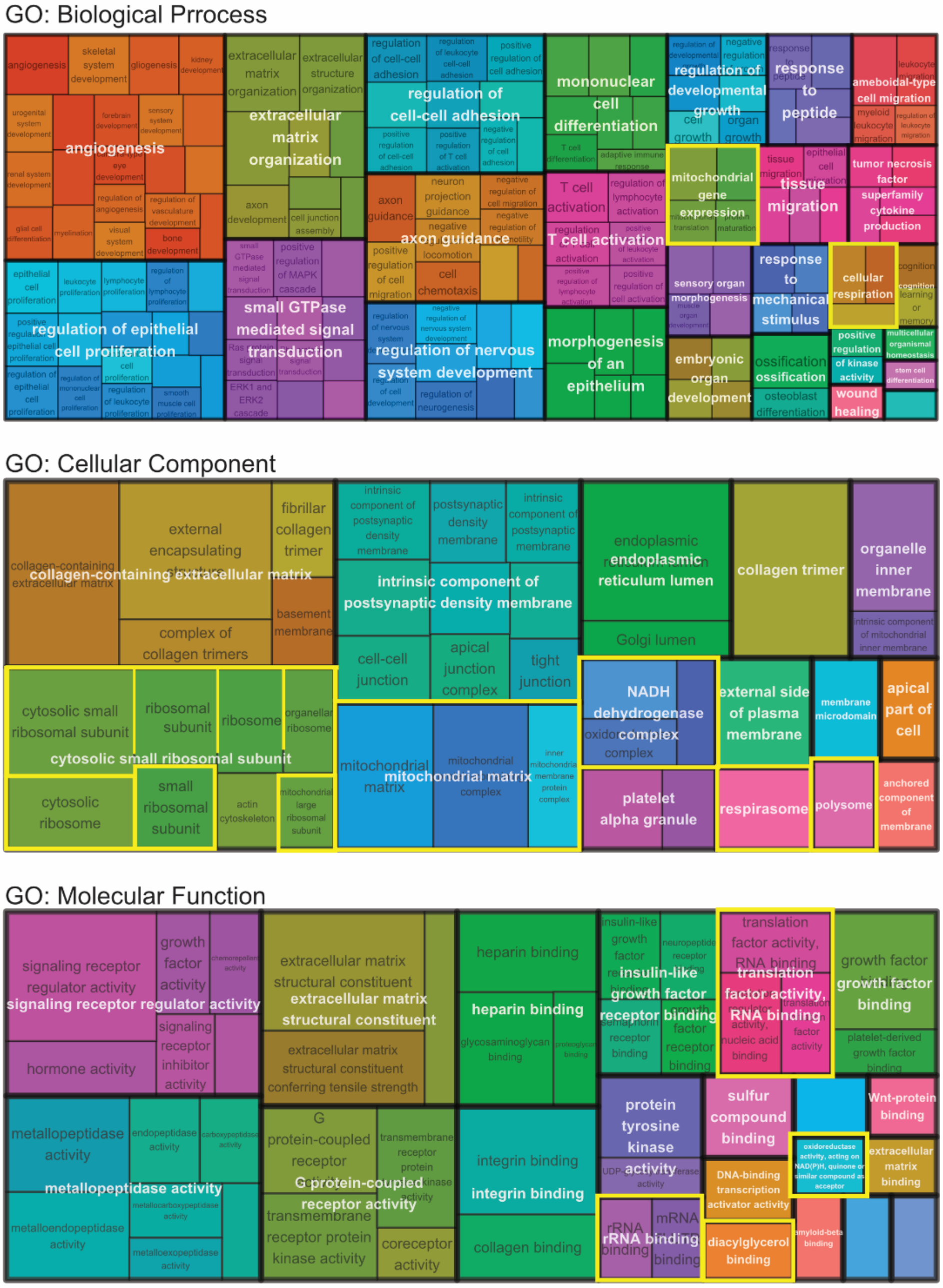

Supplemental Figure 3. Specific GO terms from the categories "Biological Process", "Cellular Component" and "Molecular Function" overrepresented in DEGs shown with REVIGO TreeMap which aims at simplifying the redundance of GO sets by grouping similar terms based on their semantic similarity (S. Sayols 2023). Upregulated terms in adult samples are highlighted with yellow boxes.

Supplemental Figure 4

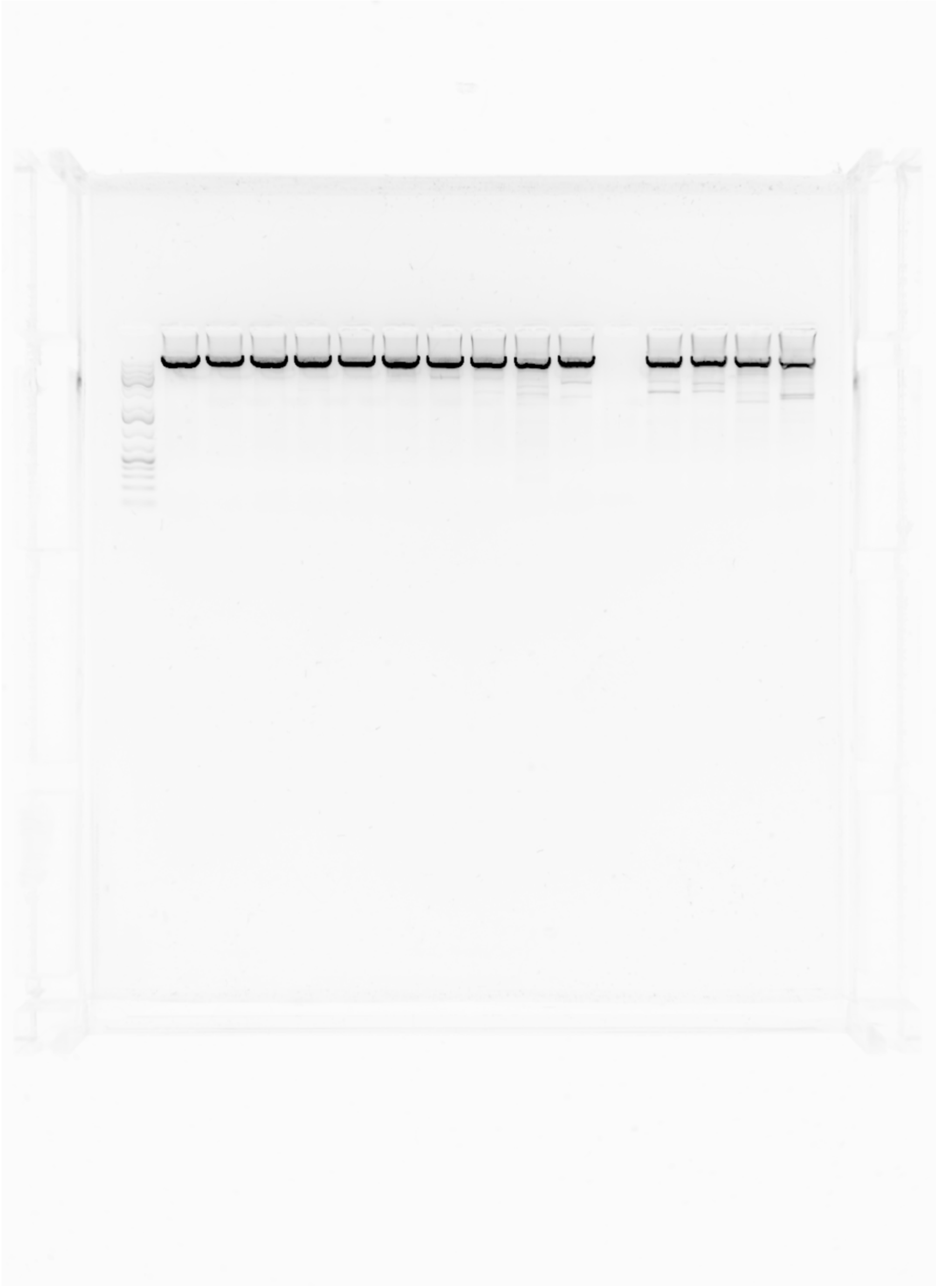

Supplemental Figure 4. Long-range PCR of the major arc of the mtDNA showing multiple mtDNA deletions in individuals older than 55 years. The original gel image displays several distinct bands, indicating the presence of mtDNA deletions of varying sizes. These deletions are characteristic of age-related mitochondrial dysfunction, commonly observed in skeletal muscle samples from elderly individuals

Supplemental Figure 5

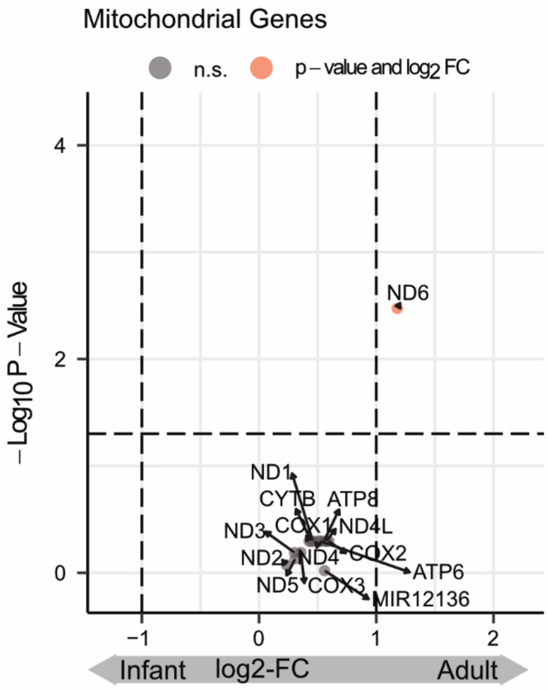

Supplemental Figure 5. RNA expression of mitochondrial genes. Volcano plot of 14 mitochondrial genes with one significantly up-regulated gene in adult skeletal muscle. The x-axis displays the log2 fold change in expression and the y-axis the negative log10 of adjusted p-value. Genes with p-value less than 0.05 and an absolute log2 fold change greater than 1 are colored red.

Supplemental Table 1: Demographic data of subjects and morphological findings in muscle biopsies

Proband	Age at biopsy (sex)	Site of muscle biopsy	Suspicion clinical diagnosis	Serum creatine-kinase (U/L)	Muscle involvement clinical/ EMG /MRI	Histology	Electron microscopy
I1	18month (f)	QFM	Epilepsy, developmental delay	94	Muscle hypotonia	Low variation in muscle fiber diameter	Unspecific
I2	4 month (f)	QFM	Developmental delay	101	Muscle hypotonia	Minor single fiber atrophy, non-specific degenerative changes	Unspecific
I3	14 month (male)	QFM	Epilepsy, developmental delay	124	Muscle hypotonia	Minor single fiber atrophy	Unspecific
I4	11 month (f)	QFM	Epilepsy, developmental delay	83	Muscle hypotonia	Moderate single fiber atrophy	Subtle mitochondrial aggregates
I5	22 month (m)	QFM	Transient motoric developmental delay	95	Muscle hypotonia	Low variation in muscle fiber diameter, mild mitochondrial aggregates	Unspecific
I6	28 month (f)	QFM	Transient gait disorder	110	none	Low variation in muscle fiber diameter	Subtle polymorph mitochondria
I7	7 month (f)	QFM	Cardiomyopathy, developmental delay	37	none	Low variation in muscle fiber diameter	Unspecific
I8	13 month (f)	QFM	Cardiomyopathy		none	Low variation in muscle fiber diameter	Unspecific
A1	30 years (m)	QFM	Chronic pain disorder	241	none	No pathology	Unspecific
A2	19 years (male)	QFM	Increase of CK of unclear etiology	1248	none	No pathology	Unspecific
A3	55 years (f)	QFM	Myalgia	182	Mild proximal weakness	No pathology	Unspecific
A4	45 years (f)	DM	Myopathy	48	Mild proximal weakness	Low variation in muscle fiber diameter	Subtle mitochondrial aggregates
A5	45 years (f)	IM	Myasthenia gravis		Inducable paralysis	c5b-9 aggregates at motoric end plates	Subtle mitochondrial aggregates
A6	44 years (f)	GM	Crampi syndrome	72	none	Low variation in muscle fiber diameter	Subtle mitochondrial aggregates, single muscle fiber atrophy
A7	35 years (m)	QFM	Dermatomyositis	182	none	Low variation in muscle fiber diameter, type 2 fiber predominance	Subtle mitochondrial aggregates
A8	40 years (m)	QFM	Axonal neuropathy		Peripheral neuropathy, no weakness	Low variation in muscle fiber diameter	Unspecific
A9	65 years (m)	QFM	Myopathy		Proximal mild weakness	Low variation in muscle fiber diameter	Single atrophic muscle fibers
A10	56 years (m)	DM	Myalgia	256	none	Moderate single fiber atrophy	Unspecific
A11	76 (m)	QFM	Myalgia	normal	none	normal	not performed
A12	76 (f)	QFM	Myalgia		none	normal	Unspecific
A13	82 (f)	QFM	Myalgia	normal	none	normal	Unspecific

A14	83 (f)	QFM	Myalgia		none	Type 2b atrophy	Unspecific

Supplemental Table 2: Analysis of skeletal muscle samples

Subject	Histological and ultrastructural re-evaluation	Mass spectrom etry	RNAseq	Mitochondrial assay (long range PCR)	Mitochondrial assay (mt copy number)	Immuno-fluorescence	Immunohisto chemistry and morphometry
I1	X	X	X				X
I2	X	X		X	X		X
I3	X	X				X	X
I4	X	X	X				X
I5	X	X		X	X		X
I6	X	X	X				X
I7	X		X				X
I8	X			X	X		X
A1	X	X	X	X	X		X
A2	X	X					X
A3	X	X	X	X	X		X
A4	X	X	X			X	X
A5	X	X		X	X		X
A6	X	X	X	X	X		X
A7	X	X	X				X
A8	X	X	X	X	X		X
A9	X	X		X	X		X
A10	X	X	X	X	X		X
A11	X			X			
A12	X			X			
A13	X			X			
A14	X			X			

Supplemental Table 3 : DEPs in adult muscle samples compared to infants (GO terms and literature search)

Protein	Name	Function	Known functional role in human skeletal muscle	Log2 Fold-change	Adjusted p-Value
**Down-regulated in adults**					
S100A1	Protein S100-A1	**Sarcomere and structural** Excitation-contraction coupling; developmental processes	Yes	-3.68	0.008
XIRP2	Xin actin-binding repeat-containing protein 2	**Sarcomere and structural** Myofibril development and regeneration; Type 2 fiber	Yes	-2.72	0.021
XIRP1	Xin actin-binding repeat-containing protein 1	**Sarcomere and structural** Myofibril development and regeneration	Yes	-1.06	0.009
FHOD1	FH1/FH2 domain-containing protein 1	**Sarcomere and structural** Assembly of F-actin structures; cell elongation	Yes	-1.03	0.029
MSN	Moesin	**Sarcomere and structural** Stabilization of plasma membrane interactions	Yes	-1.32	0.024
MAP4	Microtubule-associated protein 4	**Sarcomere and structural** Promotes microtubule assembly; Myogenesis	Yes	-1.13	0.038
MUSTN1	Musculoskeletal embryonic nuclear protein 1	**Sarcomere and structural** Musculoskeletal development and regeneration	Yes	-2.06	0.040
DTNA	Dystrobrevin alpha	**Sarcomere and structural**	Yes	-1.89	0.016

		Formation and stability of synapses			
TNXB	Tenascin-X	**Sarcomere and structural** Cell adhesion Extracellular matrix (ECM)	Yes	-1.78	0.004
AKR7A2	Aflatoxin B1 aldehyde reductase member 2	**Metabolic** Lipid metabolism	No	-2.72	0.002
AKR1C1	Aldo-keto reductase family 1 member C2	**Metabolic** Lipid and steroid metabolism	No	-2.07	0.042
OXCT1	Succinyl-CoA:3-ketoacid coenzyme A transferase 1, mitochondrial	**Metabolic** Lipid metabolism	Yes	-1.71	0.026
HK1	Hexokinase-1	**Metabolic** Glycolysis **Immune** Innate immunity	Yes	-1.41	0.013
AGK	Acylglycerol kinase, mitochondrial	**Metabolic** Lipid metabolism	Yes	-1.32	0.035
MPI	Mannose-6-phosphate isomerase	**Metabolic** Nucleotide-sugar biosynthesis	Yes	-1.24	0.033
GBE1	1,4-alpha-glucan-branching enzyme	**Metabolic** Glycogen biosynthesis	Yes	-1.20	0.026
CARNS1	Carnosine synthase 1	**Metabolic** Synthesis of carnosine and homocarnosine	Yes	-1.20	0.025
ACTR1B	Beta-centractin	**Immune** Involved in microtubule based vesicle motility	No	-2.07	0.013
CLTC	Clathrin heavy chain 1	**Immune** Clathrin-mediated endocytosis Costamere organization	Yes	-1.62	0.023
XRCC5	X-ray repair cross-complementing protein 5	**Immune** DNA repair, involved in innate immune response	Yes	-1.76	0.012

SCARB2	Lysosome membrane protein 2	**Immune** Host-virus interaction	No	-1.37	0.040
PSMC3	26S proteasome regulatory subunit 6A	**Maintenance** Cellular homeostasis	No	-1.42	0.039
PSMD7	26S proteasome non-ATPase regulatory subunit 7	**Maintenance** Cellular homeostasis	Yes	-1.25	0.049
SFPQ	Splicing factor, proline- and glutamine-rich	**Maintenance** DNA recombination; DNA repair	Yes	-1.89	0.001
COPA	Coatomer subunit alpha;Xenin;Proxenin	**Maintenance** Protein transport	Yes	-1.78	0.010
CMPK1	UMP-CMP kinase	**Maintenance** Pyrimidine biosynthesis	No	-1.71	0.026
LMO7	LIM domain only protein 7	**Maintenance** Regulator of Skeletal muscle relevant genes	Yes	-1.60	0.043
VPS26A	Vacuolar protein sorting-associated protein 26A	**Maintenance** Protein transport	No	-1.51	0.039
F13A1	Coagulation factor XIII A chain	**Maintenance** Chaperone protein	No	-1.37	0.023
TAGLN	Transgelin	**Maintenance** Muscle protein; Calcium homeostasis; Actin Cytoskeleton organization	Yes	-1.32	0.049
TCP1	T-complex protein 1 subunit alpha	**Maintenance** Chaperone protein	Yes	-1.26	0.001
CAND1	Cullin-associated NEDD8-dissociated protein 1	**Maintenance** Ubl conjugation pathway	Yes	-1.21	0.039
PCMT1	Protein-L-isoaspartate O-methyltransfer	**Maintenance** Protein repair	No	-1.21	0.009

	ase;Protein-L-isoaspartate(D-aspartate) O-methyltransferase				
HSPA5	78 kDa glucose-regulated protein	**Maintenance** Chaperone protein	Yes	-1.05	0.018
TOM1	Target of Myb protein 1	**Maintenance** Protein transport	No	-1.00	0.029
MLIP	Muscular LMNA-interacting protein	**Development** Myoblast differentiation; cardiac adaptation to stress	Yes	-1.84	0.028
KLHL40	Kelch-like protein 40	**Development** Ubl conjugation pathway	Yes	-1.18	0.022
RPS13	40S ribosomal protein S13	**Protein biosynthesis** Ribosomal protein	No	-1.87	0.035
RPL26	60S ribosomal protein L26;60S ribosomal protein L26-like 1	**Protein biosynthesis** Ribosomal protein	No	-1.44	0.041
EIF3L	Eukaryotic translation initiation factor 3 subunit L	**Protein biosynthesis**	No	-1.08	0.034
EPRS	Bifunctional glutamate/proline--tRNA ligase; Glutamate--tRNA ligase; Proline--tRNA ligase	**Protein biosynthesis** Translation regulation	No	-1.62	0.011
RARS	Arginine--tRNA ligase, cytoplasmic	**Protein biosynthesis**	Yes	-1.29	0.043
**Up-regulated in adult**					
MYL6B	Myosin light chain 6B	**Sarcomere and structural** Muscle contraction Typ1 fiber	Yes	1.78	0.044

PDLIM1	PDZ and LIM domain protein 1	**Sarcomere and structural** Cytoskeletal protein Adapter protein to cytoskeleton Type 1 fiber	Yes	1.41	0.033
LMCD1	LIM and cysteine-rich domains protein 1	**Protein biosynthesis** Transcription regulation;	Yes	1.10	0.020
DCXR	L-xylulose reductase	**Metabolic** NADH dependent glucose metabolism	No	1.07	0.049
NDUFA5	NADH dehydrogenase [ubiquinone] 1 alpha subcomplex subunit 5	**Metabolic** NADH dependent mitochondrial complex I	Yes	1.05	0.034
NDUFB10	NADH dehydrogenase [ubiquinone] 1 beta subcomplex subunit 10	**Metabolic** NADH dependent mitochondrial complex I	Yes	1.12	0.041
TMEM38A	Trimeric intracellular cation channel type A	**Metabolic** Potassium transport	Yes	1.54	0.005
USMG5	Up-regulated during skeletal muscle growth protein 5	**Metabolic** Mitochondrial membrane ATP synthesis	Yes	3.23	0.001
HBG2	Hemoglobin subunit gamma-2	**Metabolic** Oxygen transport	No	4.26	0.011
UBE2N	Ubiquitin-conjugating enzyme E2 N	**Immune** Transferase DNA repair, involved in interferon beta production, IL17A-mediated signaling pathway	No	1.72	0.024
PSMB6	Proteasome subunit beta type-6	**Maintenance** Proteolytic degradation	No	1.44	0.007
